# Investigating Correlation Between Gut Microbiota and Rheumatoid Arthritis Subtypes by Mendelian Randomization

**DOI:** 10.3390/pathogens14040385

**Published:** 2025-04-15

**Authors:** Jiaqi Wu, Yao Peng, Ruimin Tian, Hao Yu, Huating Hu, Qingchun Huang, Youhua Xu, Liang Liu, Hudan Pan

**Affiliations:** 1 School of Pharmacy, Faculty of Medicine, Macau University of Science and Technology, Macau 999078, China; jiaqiwu6998@gzucm.edu.cn (J.W.);; 2 The Second Affiliated Hospital of Guangzhou University of Chinese Medicine, Guangzhou 510006, China; 3 Macau Institute for Translational Medicine and Innovation, University of Macau, Macau 999078, China

**Keywords:** Mendelian randomization study, gut microbiome, rheumatoid arthritis, seronegative, seropositive

## Abstract

**Background**: Previous studies have demonstrated that the gut microbiota (GM) and rheumatoid arthritis (RA) are significantly associated, but the causal relationship has not been fully elucidated. **Methods**: We investigated the association between GM and RA using Mendelian randomization (MR) with two independent samples. Our study aimed to determine the causal relationship between gut microbiota and RA, including its seronegative and seropositive subtypes. Using data from a genome-wide association study (GWAS), we identified instrumental variables for 211 gut bacteria types. We then analyzed the FinnGen GWAS dataset, which included 3877 seronegative RA cases and 285,035 controls, along with 4290 seropositive RA cases and 368,362 controls, employing the inverse variance weighted (IVW) method and rigorous tests for pleiotropy and heterogeneity to ensure reliability. **Results**: The IVW results revealed that *Prevotella 9*, *Sutterella*, and *Christensenellaceae R.7* exhibited an adverse correlation with seronegative RA (*p* < 0.05). Additionally, *Lachnospira*, *Slackia*, *Roseburia*, *Barnesiella*, and *Prevotella 7* were associated with a reduced occurrence of seropositive RA (*p* < 0.05). Conversely, *Ruminococcaceae UCG002* and *Ruminococcus gauvreauii* were linked to an increased susceptibility to seropositive RA (*p* < 0.05). Notably, no significant heterogeneity (*p* > 0.05) or pleiotropy (*p* > 0.05) was detected in the analysis of the significant MR estimates. **Conclusions**: Our study suggested significant associations between several gut bacteria and RA subtypes, indicating a potential microbial influence on RA development. These findings enhance our understanding of the gut-joint axis in RA and highlight promising targets for future microbiota-based therapies.

## 1. Introduction

Rheumatoid arthritis (RA) is a chronic inflammatory autoimmune disorder primarily affecting the joints, leading to synovial tissue inflammation, cartilage degradation, and potential joint destruction [1]. Patients are typically classified into two subsets based on the presence of rheumatoid factor (RF) and anticitrullinated protein antibodies (ACPAs): seropositive RA and seronegative RA [2]. Seropositive RA accounts for about two-thirds of cases and is associated with more severe symptoms, joint damage, and higher mortality [3,4,5]. This severity is attributed to immune complexes formed by ACPAs and RF, triggering complement activation [6]. While seropositive RA is well-studied, the immunopathogenesis of seronegative RA remains unclear, and it is likely more heterogeneous [7]. Understanding the distinct genetic and environmental factors influencing these RA forms is vital for improving diagnosis and management, given their significant global health burden.

The gut microbiota (GM), often called the “forgotten inner organ” [8], plays a crucial role in the development of RA. The “gut–joint” axis describes the link between gut microbiota, gut health, and immune response in relation to RA [9]. Research suggests that RA pathogenesis may begin at mucosal surfaces, where dysbiosis triggers abnormal immune interactions, potentially affecting synovial joints [10]. Dysbiosis has been observed in preclinical RA patients with RF or ACPAs, indicating its role in disease onset [11]. Notably, studies found higher levels of *Prevotella* species, particularly *Prevotella copri*, in the intestines of preclinical RA patients, suggesting dysbiosis may precede arthritis [12]. Moreover, specific microbiota changes, such as reductions in *Haemophilus* spp. and increases in *Lactobacillus salivarius*, correlate with RA severity and autoantibody levels [13]. While these findings highlight the relationship between gut microbiota and RA, further research is needed to clarify causal relationships and identify specific biomarkers across RA subtypes.

Mendelian randomization (MR) is an epidemiological method that leverages genetic data from genome-wide association studies (GWAS) to explore causal relationships between environmental factors and diseases [14]. Specifically, MR uses genetic variants, typically single-nucleotide polymorphisms (SNPs), as instrumental variables (IVs) to simulate randomization, helping to establish causality between exposures and outcomes. MR is particularly suited for investigating the causal role of gut microbiota in RA because it addresses the limitations of traditional observational studies, such as confounding and reverse causality. In MR, genetic variants are randomly allocated at conception, meaning they are not influenced by external factors like medications or lifestyle changes, thereby mitigating the risk of biased results [15]. This is particularly important in microbiome research, where confounding factors like diet, medication, and environmental exposures can otherwise obscure the true causal relationships.

The increasing availability of large-scale GWAS summary statistics on gut microbiota and RA has significantly enhanced the power of MR studies in this area [16,17]. In this study, we utilized a two-sample MR approach to investigate the causal association between gut microbiota and both seronegative RA and seropositive RA, using the latest summary data from GWAS. The purpose was to identify specific bacterial taxa that may influence the risk of developing RA, thereby providing insights into potential microbial therapeutic targets.

## 2. Materials and Methods

### 2.1. Study Design

This research employed a two-sample MR analysis to explore the causal links between GM taxa and seronegative RA, as well as seropositive RA. To enhance the validity of the study findings and reduce the impact of extraneous factors, the MR analysis adhered to the following core assumptions [15]:

(1) The SNPs selected as IVs exhibit strong associations with gut microbiota;
(2) The IVs are independent of confounders that could introduce bias;
(3) The IVs influence the outcome solely through the exposure, ensuring no direct effects on RA beyond their association with GM.

**Figure 1:**
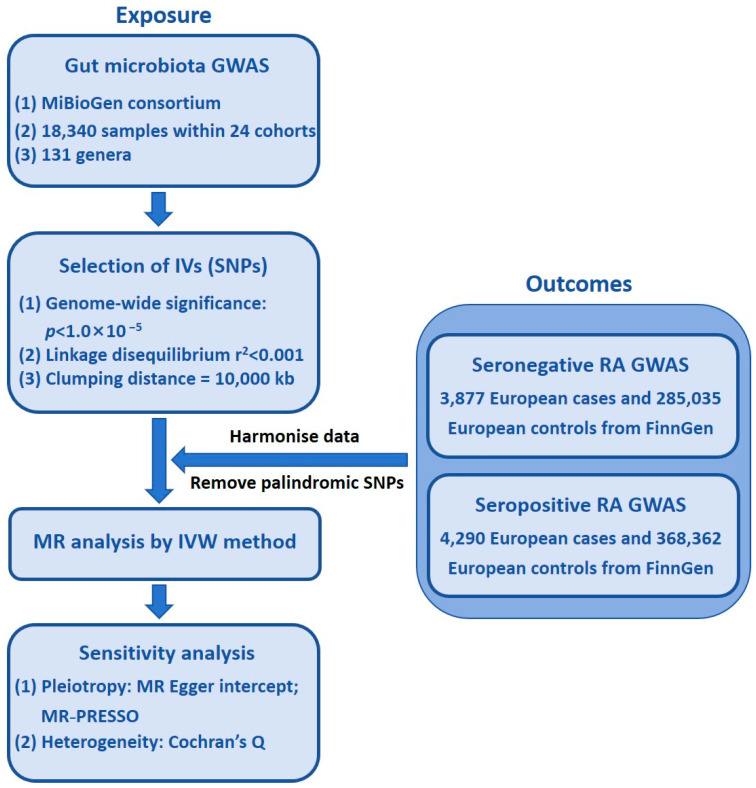
Flowchart of MR investigation. GWAS, genome-wide association study; MR, Mendelian randomization; IV, instrumental variable; IVW, inverse-variance weighted; SNP, single-nucleotide polymorphism; RA, rheumatoid arthritis.

Figure 1 displays a flowchart of the MR investigation.

### 2.2. Ethics Statement

This study utilized Mendelian randomization to analyze aggregated data from publicly available databases, specifically the MiBioGen Consortium (https://www.mibiogen.gcc.rug.nl/, accessed on 25 November 2023) and the FinnGen research consortium (https://r5.finngen.fi/, accessed on 25 November 2023). Since these datasets are publicly accessible and have already received ethical approval from the respective institutions at the time of original data collection, no further ethical approval was necessary for this re-analysis.

### 2.3. Study Population and Data Sources

The genetic summary data for GM taxa were derived from the MiBioGen consortium, a large-scale GWAS meta-analysis of gut microbiota composition across multiple cohorts, including individuals of European ancestry. The RA genetic dataset, including seronegative RA and seropositive RA subtypes, was obtained from the publicly available GWAS datasets from the FinnGen consortium. These datasets include individuals diagnosed with RA based on clinical and serological criteria.

Eligibility criteria for participants included individuals of European descent with well-characterized microbiota and RA status. The RA cases were identified through physician diagnosis, ICD-10 codes, and serological markers, ensuring accurate classification into seronegative RA and seropositive RA subtypes. The recruitment periods varied by cohort, with data collection spanning multiple years, ensuring robust representation.

### 2.4. Exposure Data

In the exposure data, we selected SNPs related to the composition of the GM in humans. These SNPs were chosen from GWAS data amassed by the international alliance known as MiBioGen (https://www.mibiogen.gcc.rug.nl, accessed on 25 November 2023). The extensive GWAS delved into the genotype details and 16S ribosomal RNA gene sequencing profiles of 18,340 individuals across 24 study groups spanning 11 nations. By scrutinizing the diversity of gut microbiome species in the populations under study, a grand total of 122,110 genetic variation sites and 211 species were pinpointed, spanning across 9 phyla, 16 classes, 20 orders, 35 families, and 131 genera.

### 2.5. Outcome Data

Based on the FinnGen research project (https://r5.finngen.fi/), we obtained GWAS summary statistics for both seronegative and seropositive RA. Specifically, the summary-level data for seronegative RA were derived from a comparison between 3877 European seronegative RA cases and 285,035 healthy European control individuals (https://storage.googleapis.com/finngen-public-data-r9/summary_stats/finngen_R9_RHEUMA_SERONEG.gz, accessed on 25 November 2023), while the data for seropositive RA were based on 4290 European seropositive RA cases and 368,362 healthy European control individuals (https://storage.googleapis.com/finngen-public-data-r9/summary_stats/finngen_R9_RHEUMA_SEROPOS_STRICT.gz, accessed on 25 November 2023). These healthy controls were recruited from the general population and are inherently incorporated into the GWAS datasets provided by the FinnGen consortium (R9 data release), thereby ensuring robust and reliable genetic association analyses.

### 2.6. Selection of IVs

Since only a small number of SNPs showed genome-wide significance with *p* < 1 × 10^−8^, a less stringent threshold of *p* < 1 × 10^−5^ was selected as possible IVs. This approach was adopted to ensure a sufficient number of IVs, which is crucial for maintaining statistical power in MR studies, especially when investigating complex and polygenic traits like GM. While we recognize that using a less stringent threshold may introduce weak instrument bias, this strategy has been commonly used in MR studies on microbiome-related traits [18,19,20].

To ensure the robustness of causal inferences regarding the association between GM and RA risk, we implemented several quality control procedures to select optimal IVs. Initially, IVs were chosen based on a linkage disequilibrium (LD) threshold of *r*^2^ < 0.001 and a distance of 10,000 kb [18]. The LD threshold of *r*^2^ measures the genetic correlation between two SNPs. It is calculated using the formula:

$$r^{2}=\frac{{\left(D\right)}^{2}}{\left(p1(1-p1))(p2(1-p2)\right)}$$

where *D* is the LD coefficient, and *p*1 and *p*2 are the allele frequencies of the two SNPs.

Subsequently, the MR-Pleiotropy RESidual Sum and Outlier (MR-PRESSO) test, implemented in the “MR-PRESSO” R package (Version 4.3.2), was employed to detect and correct for potential pleiotropic effects [21]. This method identifies outliers by assessing their contribution to residual pleiotropy, flagging SNPs that exhibit disproportionately large effects. Any identified outliers were excluded to mitigate bias and uphold the validity of MR assumptions.

Furthermore, to assess the strength of the selected IVs and minimize weak instrument bias, we calculated the F-statistics for each bacterial taxon using the following equation. In this context, represents the number of IVs, denotes the sample size, and indicates the proportion of variability in exposure explained by the IVs. IVs with an F-statistic below 10 were considered weak and subsequently excluded [22].

$$F=\frac{R^{2}(n-1-\kappa )}{(1-R^{2})\kappa }$$

In this case, κ is the number of IVs, while n represents the size of the sample, and $R^{2}$ signifies the proportion of variability in exposure elucidated by the IVs.

### 2.7. Statistical Analysis

All statistical analyses were performed using R software (Version 4.3.2). The MR analyses assessing the causal relationship between GM taxa and seronegative RA and seropositive RA were conducted using the “TwoSampleMR” R package (Version 4.3.2). A *p*-value < 0.05 was considered indicative of a potential causal effect.

(a) Handling of continuous variables

Continuous variables, such as effect sizes for SNP-exposure and SNP-outcome associations, were analyzed on the log-odds scale. Effect estimates were presented as odds ratios (ORs) with 95% confidence intervals (CIs) to facilitate interpretation. SNP-exposure effects were harmonized across datasets to ensure consistency in allele orientation.

(b) MR models and SNP weighting

For bacterial genera with a single IV, the Wald ratio method was used to estimate causal effects. For taxa with multiple IVs, various statistical approaches were applied, including the following:

- IVW analysis (fixed or random effects, depending on heterogeneity): considered the primary MR estimate;
- Weighted median analysis: provides a consistent estimate even if up to 50% of the instruments are invalid;
- MR-Egger regression: used to assess pleiotropy and provide bias-corrected causal estimates;
- Simple mode and weighted mode analyses: offer additional robustness checks.

Each SNP was weighted by the inverse of its squared standard error in the IVW model to optimize statistical efficiency and minimize bias.

(c) Handling of heterogeneity and pleiotropy

Heterogeneity across SNPs was assessed using Cochran’s Q test. If significant heterogeneity was detected (*p* < 0.05), a random-effects IVW model was applied to provide a more conservative and reliable estimate [23]. Horizontal pleiotropy was evaluated using the MR-Egger intercept test, where *p* > 0.05 indicated no significant pleiotropy.

(d) Sensitivity analyses

To validate the robustness of causal estimates derived from the IVW model, the following sensitivity analyses were conducted:

- MR-Egger intercept test: used to detect potential directional pleiotropy [24];
- Leave-one-out analysis: performed to evaluate whether any single SNP disproportionately influenced the results.

## 3. Study Results

### 3.1. IVs in GM Taxa

We applied stringent criteria, including genome-wide significance threshold (*p* < 1 × 10^−5^), harmonization, F-statistics, the LD test, and the MR-PRESSO test, to pinpoint a range of 3 to 22 SNPs as potential proxies for the 196 bacterial taxa under investigation. We excluded any SNP identified as an outlier in the MR-PRESSO test (*p* < 0.05). All remaining SNPs had F-statistic values of ˃10, which indicated that the strength of the associations of the IVs with the corresponding bacterial taxa was sufficient. The final compilation of selected SNPs and pertinent statistical details is presented in Supplementary Table S1.

### 3.2. Causal Effect of GM on Seronegative RA

**Figure 2:**
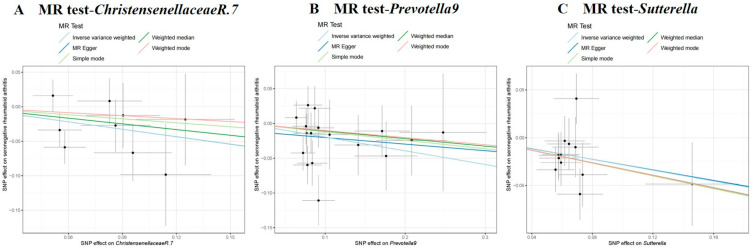
Results regarding causal associations of GM with seronegative RA risk. Points represent SNP effects on *Christensenellaceae R.7* (**A**), *Prevotella 9* (**B**), *Sutterella* (**C**), and seronegative RA. MR, Mendelian randomization; SNP, single-nucleotide polymorphism; RA, rheumatoid arthritis.

The IVW test findings indicated that a genetically predicted higher relative abundance of *Prevotella 9* (*OR* = 0.821, 95% *CI* = 0.699–0.965, *p* = 0.017), *Sutterella* (*OR* = 0.753, 95% *CI* = 0.602–0.943, *p* = 0.013), and *Christensenellaceae R.7* (*OR* = 0.696, 95% *CI* = 0.515–0.940, *p* = 0.018) were linked to a lower risk of seronegative RA (refer to Table 1 and Figure 2). To ensure the robustness of our results, we conducted Cochran’s Q test, which showed no significant heterogeneity across the SNPs (*p* > 0.05). The MR-Egger intercept analysis further indicated the absence of horizontal pleiotropy (*p* > 0.05, Supplementary Table S2). Additionally, leave-one-out sensitivity analysis confirmed that no single SNP disproportionately influenced the findings (Supplementary Figure S1).

### 3.3. Causal Effect of GM on Seropositive RA

**Figure 3:**
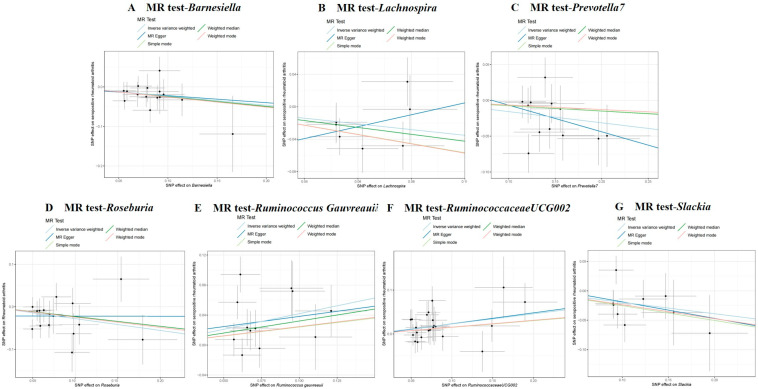
Results regarding causal associations between GM and seropositive RA risk. Points represent SNP effects on *Barnesiella* (**A**), *Lachnospira* (**B**), *Prevotella 7* (**C**), *Roseburia* (**D**), *Ruminococcus gauvreauii* (**E**), *Ruminococcaceae UCG-002* (**F**), *Slackia* (**G**), and seropositive RA. MR, Mendelian randomization; SNP, single-nucleotide polymorphism; RA, rheumatoid arthritis.

Analyses revealed *Ruminococcaceae UCG-002* (*OR* = 1.258, 95% *CI* = 1.070–1.480, *p* = 0.006) and *Ruminococcus gauvreauii* (*OR* = 1.519, 95% *CI* = 1.161–1.988, *p* = 0.002) to have an association with a higher seropositive RA risk (Table 1 and Figure 3). Conversely, it revealed a genetically predicted higher abundance of *Lachnospira* (*OR* = 0.703, 95% *CI* = 0.504–0.981, *p* = 0.038), *Slackia* (*OR* = 0.823, 95% *CI* = 0.685–0.987, *p* = 0.036), *Roseburia* (*OR* = 0.757, 95% *CI* = 0.608–0.943, *p* = 0.013), *Barnesiella* (*OR* = 0.793, 95% *CI* = 0.651–0.966, *p* = 0.021), and *Prevotella 7* (*OR* = 0.855, 95% *CI* = 0.760–0.962, *p* = 0.009) to have an association with a reduced seropositive RA risk (Table 1 and Figure 3). To ensure the validity of our assumptions, Cochran’s Q test revealed no significant heterogeneity (*p* > 0.05, Table 1), while the MR-Egger intercept test indicated no evidence of horizontal pleiotropy (*p* > 0.05, Supplementary Table S3). Furthermore, leave-one-out sensitivity analysis confirmed the stability of our findings (Supplementary Figure S2).

## 4. Discussion

A significant finding of this study is the identification of *Prevotella* as the primary bacterial genus associated with dysbiosis in both seropositive and seronegative subtypes of RA. *Prevotella* represents a diverse group of gram-negative anaerobic bacteria, comprising over 50 known species. Many of these species play context-dependent roles in maintaining the balance between health and disease [25].

The genus Prevotella has garnered increasing attention in RA research, yet its role remains complex and, at times, contradictory. Several studies have reported that an increased abundance of *Prevotella*, particularly *Prevotella copri*, is associated with symptomatic RA and an increased risk of disease development, especially among individuals with genetic susceptibility [11,26]. *Prevotella copri* is the most frequently studied species in the context of RA due to its enriched presence in the fecal microbiota of patients [27,28] and its occasional detection in synovial fluid [29]. In murine models, fecal microbiota transplantation from RA patients with high *Prevotella copri* abundance induced a TH17-mediated proinflammatory response and RA-like phenotype in genetically susceptible mice [30]. Correspondingly, RA patients have been found to produce TH1 and TH17 responses to *Prevotella copri*-specific antigens, further supporting its potential pathogenic role [31]. However, this genus also includes species with anti-inflammatory properties. For example, *Prevotella histicola*, isolated from the human gut microbiome, has been shown to reduce the incidence of collagen-induced arthritis in HLA-DQ8 transgenic mice by modulating immune responses and suppressing proinflammatory cytokines such as TNF-α, IL-2, and IL-17 [32]. This apparent dichotomy within the genus *Prevotella* may be attributable to species-level or even strain-level genetic variability, leading to divergent immunomodulatory effects.

Notably, in our study, *Prevotella* was found to be associated with both seropositive and seronegative RA, raising the possibility that different *Prevotella* species or strains may exert distinct immunological influences across RA subtypes. Seropositive RA is typically characterized by more pronounced autoimmunity and systemic inflammation, whereas seronegative RA may involve alternative pathogenic pathways, potentially mediated by different microbial or environmental triggers. It is plausible that *Prevotella copri* might contribute more directly to the pathogenesis of seropositive RA via antigenic mimicry or immune priming, while other species like *Prevotella* histicola might have a protective or regulatory role. These findings underscore the necessity of species- and strain-level resolution in future microbiome analyses to disentangle the complex and potentially bidirectional roles of *Prevotella* in RA. Further mechanistic studies, incorporating metagenomic sequencing, host-microbe interaction models, and immune profiling, are warranted to delineate the causal pathways by which different *Prevotella* species modulate RA risk and to clarify their relevance across clinical subtypes.

Notably, a higher abundance of Sutterella was associated with a reduced risk of developing seronegative RA, aligning with previous findings in RA research [33,34]. This suggests that Sutterella may play a protective role, and its depletion could contribute to disease onset through mechanisms yet to be fully understood.

Interestingly, our analysis revealed that Ruminococcus gauvreauii was positively associated with an increased risk of seropositive RA. Although direct evidence is limited, this species has been linked to other immune-mediated diseases, such as spondyloarthritis and Crohn’s disease, possibly through effects on mucosal immunity and intestinal barrier integrity [35]. A plausible mechanism involves its role in modulating T-cell polarization, particularly the Th17 pathway, which is critically involved in RA pathogenesis. *Ruminococcus gauvreauii* is a known butyrate-producing bacterium, and butyrate has been shown to regulate the balance between regulatory T cells (Tregs) and Th17 cells, an axis central to autoimmune regulation [36]. While butyrate generally promotes immune tolerance via Treg induction, under certain conditions, it may also contribute to Th17-driven inflammation. Recent MR studies have also supported a causal relationship between gut microbiota composition, including *Ruminococcus gauvreauii* group, and Th17 activation, suggesting that *Ruminococcus gauvreauii* might influence systemic immune responses through this pathway [37]. This Th17-skewed immune modulation may be particularly relevant to the heightened inflammatory state in seropositive RA. However, mechanistic studies are needed to clarify the species-specific role of *Ruminococcus gauvreauii* in RA and its potential as a therapeutic target.

We also found that *Lachnospira* was inversely associated with seropositive RA risk. A 2024 study similarly reported its downregulation in new-onset RA patients and a negative correlation with rheumatoid factor IgG (RF-IgG) levels [38]. This supports the hypothesis that *Lachnospira* may modulate RA progression through immune or metabolic regulation.

Finally, *Roseburia* emerged in our analysis as a protective factor against seropositive RA, consistent with findings by Wu et al. [39], who reported an inverse relationship between *Roseburia* abundance and RA disease activity markers such as erythrocyte sedimentation rate and RF-IgM levels. Together, these taxa highlight the complex, subtype-specific interplay between gut microbiota and RA pathogenesis, emphasizing the need for species-level investigations and functional validation.

This study revealed that the GM taxa associated with seronegative RA differ from those associated with seropositive RA, emphasizing the distinct pathogeneses of these two RA subtypes. Notably, *Prevotella* was significantly associated with both seronegative and seropositive RA. However, the mechanisms through which different Prevotella subgenera contribute to these subtypes remain unclear. The conflicting findings in the literature suggest that certain *Prevotella* species, such as *Prevotella histicola*, may have protective effects by promoting Treg differentiation and inhibiting Th17 responses. In contrast, *Prevotella copri* has been linked to a pro-inflammatory Th17 response, contributing to RA. These differences may be influenced by host genetic factors, microbial metabolites like short-chain fatty acids, or host-microbe interactions. Further studies are needed to explore how genetic, metabolic, and immune factors interact to determine the pathogenic or protective roles of *Prevotella* in RA.

This study has several limitations. First, the GWAS data used were exclusively from individuals of European ancestry, which may limit the generalizability of our findings to other ethnic groups with different genetic backgrounds and microbial compositions. Genetic variability can influence gut microbiota and immune responses, potentially affecting the gut microbiota-RA relationship. Differences in host genetics, such as variations in immune system-related genes (e.g., HLA-DRB1), may shape microbial communities and alter disease susceptibility. Therefore, future studies incorporating multi-ethnic cohorts and stratified analyses based on genetic background are warranted to validate these associations and to better elucidate gene–microbiota interactions in RA pathogenesis. Second, the GM data were based on 16S rRNA gene sequencing, which limits taxonomic resolution to the genus level and precludes species- or strain-level identification. This may obscure important functional differences between closely related taxa and lead to underestimation or misclassification of causally relevant microbes. High-resolution metagenomic sequencing should be employed in future studies to capture more precise microbial signatures. Third, although MR is a powerful tool for inferring potential causality, it relies on core assumptions—such as no horizontal pleiotropy and the validity of instrumental variables—which may not fully account for the complexity of host–microbiome interactions. Additionally, it cannot entirely exclude the possibility that some microbial alterations are secondary to RA pathophysiology. Thus, while our findings support a directional relationship from gut microbiota to RA, interpretations should remain cautious. Longitudinal studies or analyses of preclinical RA cohorts are needed to confirm whether microbial shifts precede disease onset. 

To further validate the causal relationship between GM and RA risk, future research should integrate multi-ethnic GWAS data to enhance the applicability of findings across populations. Combining metagenomic and metabolomic profiling will also enable more accurate identification of species-level taxa and their functional contributions. Moreover, in vivo and in vitro mechanistic studies are essential to elucidate gut-joint immune pathways, such as Treg/Th17 balance and cytokine-mediated inflammation, that may mediate the impact of gut microbiota on RA development. Ultimately, identifying specific bacterial taxa implicated in RA could provide new insights into disease pathogenesis and open avenues for microbiome-based therapeutic strategies.

In conclusion, this Mendelian randomization study provides genetic evidence supporting potential causal associations between gut microbiota and both seronegative and seropositive rheumatoid arthritis. While the causality inferred from MR should be interpreted with caution, our findings are consistent with prior observational studies and contribute to the growing body of evidence linking gut microbiota to RA pathogenesis. The identified taxa may serve as potential biomarkers, offering new insights into disease mechanisms and guiding future research toward more effective management and prevention strategies for RA. Moreover, these findings suggest that microbiota-based therapies, such as probiotics or dietary interventions targeting specific gut taxa, could be explored as potential therapeutic strategies for RA. For example, targeting *Prevotella* species or other identified taxa with a protective role could help restore immune homeostasis and potentially reduce RA risk or severity. Further studies integrating multi-omics approaches and functional validation are warranted to strengthen these associations, better understand their underlying mechanisms, and evaluate their therapeutic potential in clinical settings.

## Figures and Tables

**Table 1:** Significant results for MR analysis of the samples and sensitivity analysis.

Traits (Outcome)	Taxonomies	GM	Significant Results for MR Analysis of the Samples							Analysis of Sensitivity and Potential Pleiotropy Detection					
			MR Method	No. SNP	Beta	SE	*p* Value	OR	95 %*CI*	Method	*Q*	*p*	Egger-Intercept	*p*	MR-PRESSO
Seronegative RA	Genus	*Prevotella9*	IVW	17	−0.197	0.083	0.017	0.821	0.699–0.965	IVW	17.652	0.345	−0.011	0.648	0.417
										MR Egger	17.400	0.295			
	Genus	*Sutterella*	IVW	12	−0.283	0.114	0.013	0.753	0.602–0.943	IVW	9.066	0.616	−5.07 × 10^4^	0.988	0.619
										MR Egger	9.066	0.526			
	Genus	*Christensenellaceae R.7*	IVW	9	−0.363	0.153	0.018	0.696	0.515–0.940	IVW	7.987	0.435	−1.53 × 10^4^	0.997	0.454
										MR Egger	7.987	0.334			
Seropositive RA	Genus	*RuminococcaceaeUCG002*	IVW	22	0.230	0.083	0.006	1.258	1.070–1.480	IVW	16.643	0.733	−0.002	0.926	0.744
										MR Egger	16.634	0.677			
	Genus	*Ruminococcus gauvreauii*	IVW	12	0.418	0.137	0.002	1.519	1.161–1.988	IVW	18.500	0.071	0.011	0.812	0.105
										MR Egger	18.390	0.049			
	Genus	*Lachnospira*	IVW	7	−0.352	0.170	0.038	0.703	0.504–0.981	IVW	6.359	0.384	−0.069	0.272	0.410
										MR Egger	4.839	0.436			
	Genus	*Slackia*	IVW	9	−0.195	0.093	0.036	0.823	0.685–0.987	IVW	8.207	0.413	0.009	0.855	0.453
										MR Egger	8.165	0.318			
	Genus	*Roseburia*	IVW	16	−0.278	0.112	0.013	0.757	0.608–0.943	IVW	16.201	0.369	−0.022	0.383	0.369
										MR Egger	15.314	0.357			
	Genus	*Barnesiella*	IVW	15	−0.232	0.101	0.021	0.793	0.651–0.966	IVW	7.513	0.913	−0.004	0.901	0.906
										MR Egger	7.497	0.875			
	Genus	*Prevotella7*	IVW	12	−0.157	0.060	0.009	0.855	0.760–0.962	IVW	11.882	0.373	0.031	0.575	0.405
										MR Egger	11.497	0.320			

GM, gut microbiota; IVW, Inverse variance weighted; MR, Mendelian randomization; RA, rheumatoid arthritis; SE, Standard error; SNPs, specific single-nucleotide polymorphisms.

## Data Availability

The study contains unique findings that are published in the article/Supplementary Materials. For additional information, please contact the corresponding author directly.

## Supplementary Materials

The following supporting information can be downloaded at: https://www.mdpi.com/article/10.3390/pathogens14040385/s1.
