# Supplementary material for: Investigating Correlation Between Gut Microbiota and Rheumatoid Arthritis Subtypes by Mendelian Randomization
Source: Pathogens. 2025 Apr 15;14(4):385. doi: 10.3390/pathogens14040385 (PMC12030498; doi:10.3390/pathogens14040385)
Supplement: Supplementary file 1 [file pathogens-14-00385-s001.zip › Supplementary figure.pdf]

A MR test-*Prevotella9*

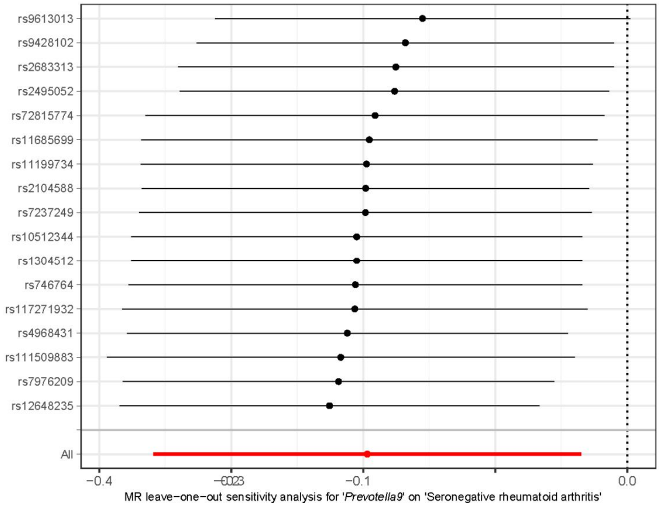

B MR test-*Sutterella*

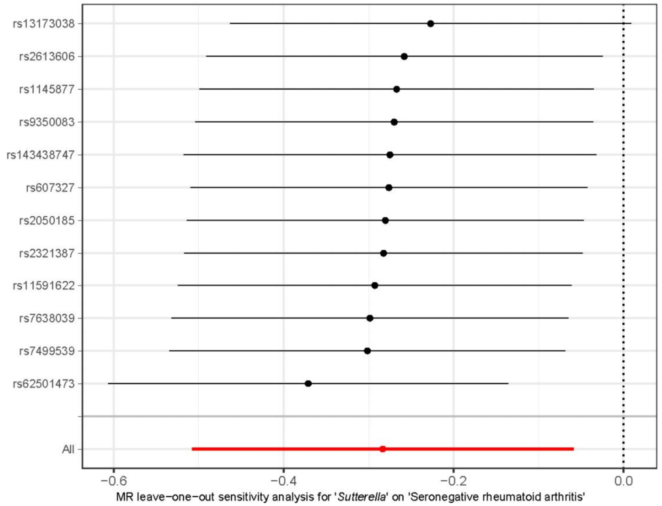

C MR test-*ChristensenellaceaeR.7*

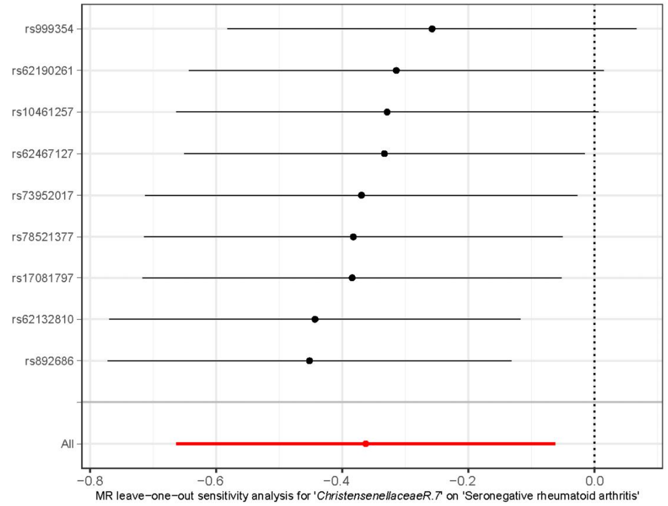

Supplementary figure S1 The leave-one-out results of GM and SNRA.GM, gut microbiota; SNRA, seronegative rheumatoid arthritis. **(A)** The leave-one-out analysis for genus-*Prevotella9* on SNRA. **(B)** The leave-one-out analysis for genus-*Sutterella* on SNRA. **(C)** The leave-one-out analysis for genus-*ChristensenellaceaeR.7* on SNRA.

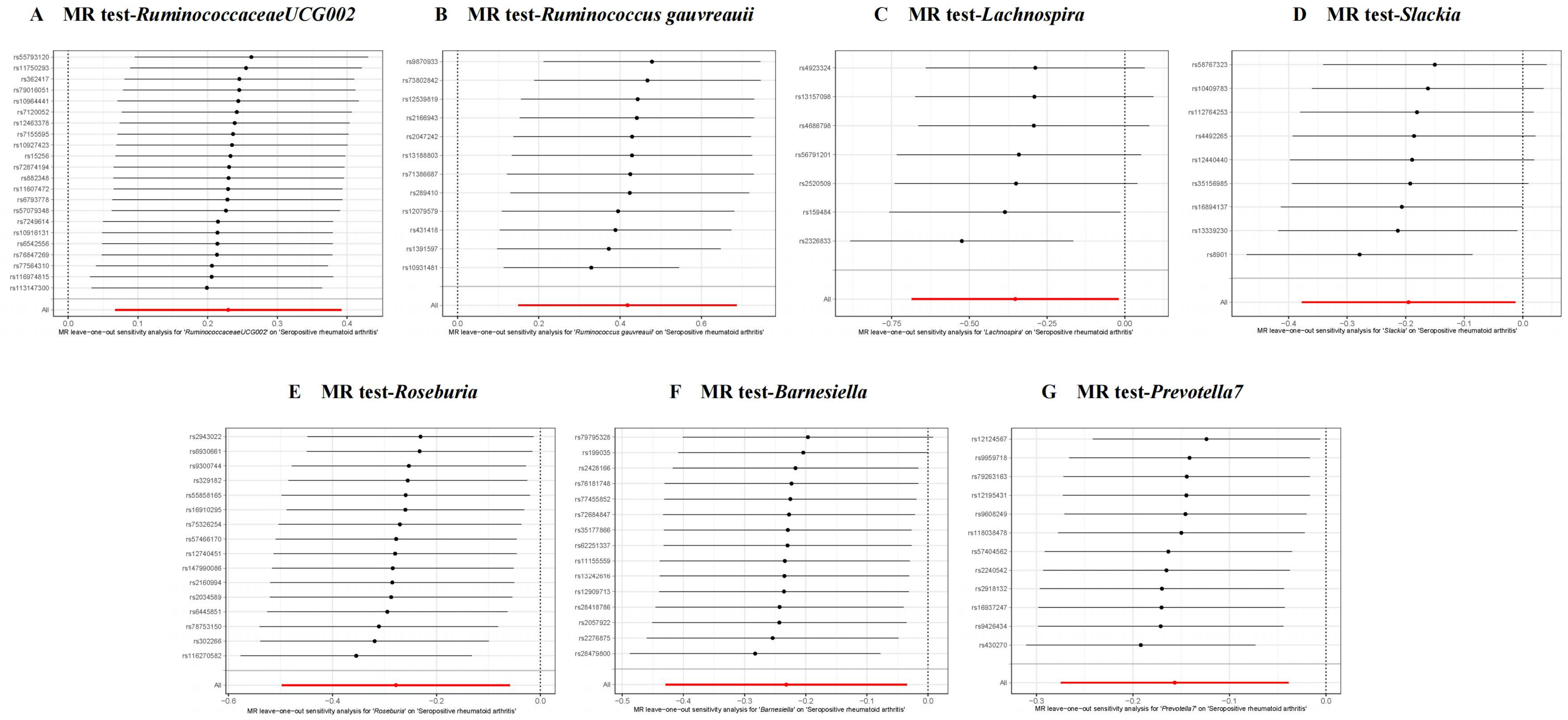

Supplementary figure S2 The leave-one-out results of GM and SPRA. GM, gut microbiota; SPRA, seropositive rheumatoid arthritis. **(A)** The leave-one-out analysis for genus-*RuminococcaceaeUCG002* on SPRA. **(B)** The leave-one-out analysis for genus-*Ruminococcus Gauvreauii* on SPRA. **(C)** The leave-one-out analysis for genus-*Lachnospira* on SPRA. **(D)** The leave-one-out analysis for genus-*Slackia* on SPRA. **(E)** The leave-one-out analysis for genus-*Roseburia* on SPRA. **(F)** The leave-one-out analysis for genus-*Barnesiella* on SPRA. **(G)** The leave-one-out analysis for genus-*Prevotella7* on SPRA.
